# Physiologically Based Pharmacokinetic (PBPK) Model for Biodistribution of Radiolabeled Peptides in Patients with Neuroendocrine Tumours

**DOI:** 10.7508/aojnmb.2016.02.005

**Published:** 2016

**Authors:** Radovan Gospavic, Peter Knoll, Siroos Mirzaei, Viktor Popov

**Affiliations:** 1 Ascend Technologies Ltd, Eastleigh, UK; 2 Faculty of Civil Engineering, University of Belgrade, Belgrade, Serbia; 3 Institute of Nuclear Medicine with PET-Center, Wilhelminenspital, Vienna, Austria

**Keywords:** Lu-177 DOTATATE, PBPK model, Radiolabeled peptides, Whole body scintigraphy

## Abstract

**Objective(s):**

The objectives of this work was to assess the benefits of the application of Physiologically Based Pharmacokinetic (PBPK) models in patients with different neuroendocrine tumours (NET) who were treated with Lu-177 DOTATATE. The model utilises clinical data on biodistribution of radiolabeled peptides (RLPs) obtained by whole body scintigraphy (WBS) of the patients.

**Methods:**

The blood flow restricted (perfusion rate limited) type of the PBPK model for biodistribution of radiolabeled peptides (RLPs) in individual human organs is based on the multi-compartment approach, which takes into account the main physiological processes in the organism: absorption, distribution, metabolism and excretion (ADME). The approach calibrates the PBPK model for each patient in order to increase the accuracy of the dose estimation. Datasets obtained using WBS in four patients have been used to obtain the unknown model parameters. The scintigraphic data were acquired using a double head gamma camera in patients with different neuroendocrine tumours who were treated with Lu-177 DOTATATE. The activity administered to each patient was 7400 MBq.

**Results:**

Satisfactory agreement of the model predictions with the data obtained from the WBS for each patient has been achieved.

**Conclusion:**

The study indicates that the PBPK model can be used for more accurate calculation of biodistribution and absorbed doses in patients. This approach is the first attempt of utilizing scintigraphic data in PBPK models, which was obtained during Lu-177 peptide therapy of patients with NET.

## Introduction

For the development of new therapeutic agents and drugs Physiologically Based Pharmacokinetic (PBPK) models have been used as a tool for the estimation of the biodistribution and toxicity of different chemical compounds and macromolecules in the living organisms (4-1). The results obtained from such models have been used in the pharmaceutical industry to assess the time course of the drugs in targeted tissues and for toxicity analysis and estimation in the case of the non-targeted tissues in living organisms (5, 6). The PBPK models have also been used in environmental and risk assessment applications (9-7). The main area of interest and research in PBPK modelling is to determine and analyse the fate of drugs, macromolecules or other chemical compounds administrated externally into living organisms. The four basic physiological processes in the living organisms which determine fate of the drugs and chemicals administrated into organisms are: absorption, distribution, metabolism and excretion (ADME) (1,2).

PBPK models consist of compartments which represent organs, tissues, blood, and these compartments are connected by blood circulation. The pharmacokinetic in living organisms is very complex and depends on physical and chemical properties of drugs and macromolecules, blood flow, physical and chemical characteristics of tissues and organs, tissue composition, permeability of various tissue membranes (10). In many cases experimental data is only available for animals and interspecies scaling could be used to extrapolate PBPK model from animals to humans (11).

In the last two decades there has been an increase of radionuclide treatment of cancer. The regulations on patient safety require adherence to standards such as calculation of biodistribution and absorbed patient dose. Also, the toxicity to healthy organs cannot be neglected and has to be considered (12). For example, for patients treated with radionuclide labeled somatostatin analogs, the renal absorbed dose plays a major role in further patient specific optimized therapy dose estimation (13), which can be calculated from sequential scintigraphic images with gamma cameras. The recent guidelines describe the good practice of clinical dosimetry reporting and proper dosimetric assessment of nuclear medicine diagnostic and therapeutic agents (14,15).

An approach employing the PBPK can be used to determine the appropriate therapeutic dose in cancer patients (16). One aspect that reduces the accuracy of these estimates is the physiological variability in patients. Another aspect is that in cancer patients this variability may be even higher since organs may be affected to different levels depending on the type of cancer and in some cases as a side effect of the applied chemotherapy. Stabin et al. has argued that the combined uncertainties in any given radiopharmaceutical dose estimate are a factor of 2 or higher, mainly because of human variability which can be increased due to disease (17).

The clinical data on biodistribution of therapeutic drugs in human patients could be of practical importance for the development of the PBPK models to improve cancer therapy.

In this work a blood flow restricted PBPK approach has been implemented and tested in patients with different neuroendocrine tumours who were treated with Lu-177 DOTATATE. The main assumption in the blood flow restricted type of PBPK model is that the considered tissue or organ could be approximated as a well-stirred compartment (18,19). In some cases the PBPK models are used in combination with in vitro data prior to the in vivo experiments in order to predict the plasma and tissue pharmacokinetics of a drug (1). Most of the previous work on PBPK models have focused on determining the model parameters for certain species.

In the present approach calibration of the PBPK model is carried out for each patient. The calibration is carried out using clinical data on the biodistribution of RLPs obtained by whole body scintigraphy of the patients.

The model is expected to provide increased accuracy when selecting the radiation dose. One reason for this is that the model considers the whole injected dose and applies the mass conservation taking into account the distribution of the radiopharmaceutical in the body and its clearance, based on information about the human physiology. In this way the time-dependent activity can be obtained for different tissues, which can further be used to determine the average absorbed dose per tissue/organ. In this way one can appropriately select the radiation dose in order to balance the risks and the benefits.

Another potential application, which was not implemented in this work, would be to monitor the patient’s condition through a comparison of the model predictions and the clinical data on the biodistribution of RLPs. Large disagreements of the model and the clinical data might indicate change in the patient’s condition.

To solve the optimization problem and to obtain the unknown model parameters the trust region method has been utilized. To speed up the numerical procedure the Broyden-Fletcher-Goldfarb-Shanno (BFGS) method has been used (23-20).

## Methods

In this study the scintigraphic data were used, which were acquired with a double head gamma camera (Symbia T6, Siemens, Erlangen, Germany) in patients with different neuroendocrine tumours who were treated with Lu-177 DOTATATE (Lutathera ®, AAA, France). The activity administered to each patient was 7400 MBq (24).

**Figure 1:**
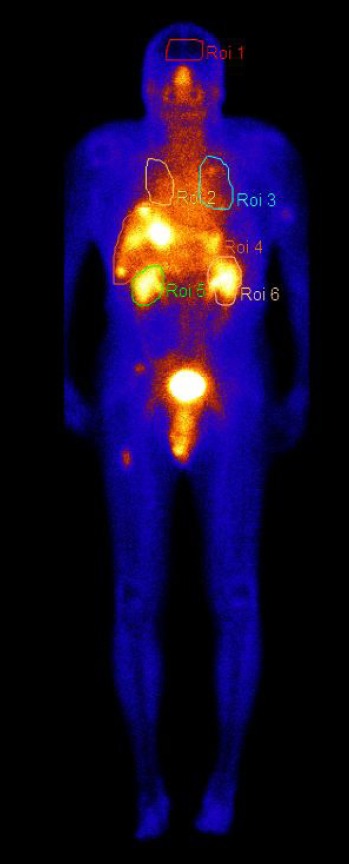
Whole body image showing radiotracer distribution in different regions of interests (ROI) over brain, lungs, kidneys and liver

It has been reported that the kidney is the dose-limiting organ in the RLPs therapies. In order to reduce the uptake of RLPs in the kidneys, each patient received an intravenous injection of a fixed combination of amino acids arginine and lysine, as recommended previously by several studies (25-27). Planar whole body imaging was performed at 0.5, 4, 24, 48 h and/or 72 h post injection for all the patients. The first whole body image was performed with full bladder and was used for normalization. Figure 1 shows WBS image with regions of interest (ROI): brain, lungs, kidneys and liver.

### Mathematical Model

### PBPK Modelling

Radiation dose estimates for radiopharmace-uticals are of importance in cancer therapy applications. Any improvement in the accuracy of the estimate would lead to a better balance between the risks and benefits of the therapy.

The average absorbed dose in target tissue is estimated as:


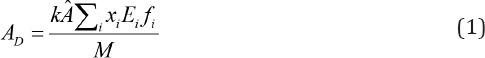


where *A_D_* is mean absorbed dose (Gy) to a target tissue; Ȃ is the time–activity integral within the target tissue (Bq s); *x_i_* is the number of radiations with energy *E_i_* per nuclear transition; *E_i_* is the mean energy of the *i*-th radiation (MeV); *f_i_* is the fraction for the *i*-th radiation absorbed in target tissue; *M* is the mass of the target tissue (kg); k is a constant (Gy kg Bq^-1^ s^-1^ MeV^-1^).

The time-activity integral Ȃ is calculated as an integral of the time-dependent activity *A* [Bq]


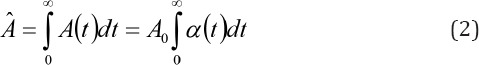


where *A*_0_ is the activity administered at time *t*=0, and α(*t*) is the fraction of the administered activity present within the source region at time *t*. In this work the average absorbed dose is not calculated as the main focus is on PBPK model development for determining the time-dependent activity *A*. Once *A* is available, it is straightforward to calculate the time-activity integral and the average absorbed dose in a tissue.

**Figure 2:**
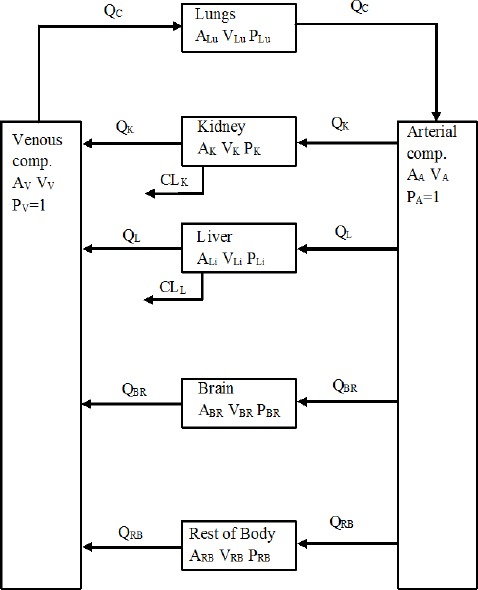
Schematic diagram for multi compartment PBPK model for biodistribution of Lu-177 DOTATATE in human body

The blood flow restricted type of the PBPK model with seven compartments was developed and was used for prediction of biodistribution and accumulation in different organs in the human body. The schematic diagram of the PBPK model with different compartments is shown in Figure 2. The nomenclature used in the Figure 1 is summarised in Table 1.

**Table 1:** Nomenclature used for the PBPK model

Q_C_ [ml/min]	Total Cardiac output
Q_T_ [ml/min]	Blood flow for individual tissue/organ
V_T_ [ml]	Volume of the tissue/organ
A_T_ [Bq/ml]	activity in tissue/organ
P_T_	Tissue to blood partition coefficients
CL_T_ [Bq/min]	Clearance or metabolic parameter

Lu: Lung, K: Kidney, L:Liver, RB: Rest of Body,

A:Arterial compartment, V: Venous compartment, BR: brain

To calibrate the model and find unknown model parameters the clinical results for four patients obtained by WBS have been used. The developed PBPK model includes the following organs: lungs, kidney, liver, brain, venous and arterial compartments and the rest of body. The results from the WBS include the whole body; however, results suggest that biodistribution occurs mainly in the liver and kidneys.

The main assumptions in the model are: (i) each tissue is modelled as homogenous, (ii) the concentration of radiopharmaceuticals in the venous blood is in equilibrium with the concentration in the tissue and the model parameters (blood flow, tissues volumes and tissue to blood partition coefficients) are considered to be constant over time.

In the PBPK model, see Figure 2, due to mass conservation the following constraint related to blood flow must be satisfied:


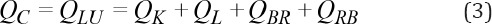


The balance equation for the case of flow perfusion-limited model for each non-clearing organ, which are the organs that do not remove/extract, except for the case of lungs and arterial and venous compartments, is given as (29, 28):


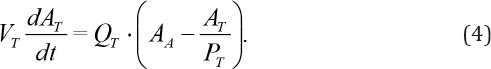


The balance equations for lungs, arterial and venous compartment are given in the following form (29, 28):


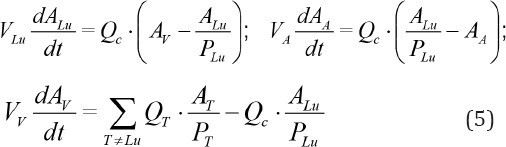


For the case of organs with clearing or extraction, e.g. kidney, or metabolic processes, e.g. liver, the balance has been expressed in terms of intrinsic clearance CL by the following relation (29, 28):


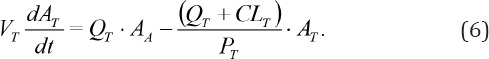


For the blood flow restricted type of models the unknown parameters, which depend on the type of drug, the macromolecule of compound, and the individual organ/tissue, include: partition coefficients P_T_ (which are responsible for absorption and distribution in the organs/tissues), for the case of tissue without clearance, and partition coefficients P_T_ and clearance CL [ml/min], for the case of tissue with clearance. The tissue/blood partition coefficient is defined as the ratio of concentration of radiopharmaceutical in tissue to that in the emergent venous blood of the tissue. The partition coefficients for chemicals/small molecules can be estimated from in vitro and/or in vivo methods (30).

It has been adopted that the average density of all organs/tissues is approximately 1 g/ml (1.056 g/ml). Cardiac output per body weight for humans is estimated to 15 [L/h·kg^0.74^] (7, 9, 31).

The mass of organs and fraction of blood flow for different organs in humans used to optimize the PBPK model are shown in Table 3e (7,9,31). Because the Blood Brain Barrier (BBB) restricts the transport of the large molecules from CBV to interstitial and cellular space of the Central Nervous System (CNS) it has been assumed that biodistribution of RLPs in the brain occurs only in CBV (33, 32). According to this assumption, the available volume/mass of the brain in the PBPK model for biodistribution is 3.8 % of total brain volume/mass, and is negligible for further measurements.

**Table 2:** Clinical results obtained by WBS for four different patients (Datasets 1 - 4). All quantities related to activity in specific organs are expressed in counts/sec (Bq)

Dataset 1				Dataset 2			
time [h]	Liver	Kidney	Brain	time [h]	Liver	Kidney	Brain
0	0	0	0	0	0	0	0
0.5	13.70	3.38	0	0.5	2.41	3.60	0.05
2.5	18.70	2.30	0	3.5	1.38	1.73	0.2
24.5	17.90	1.93	0	23.5	0.98	1.50	0.15
119.5	10.00	1.12	0	119.5	0.33	0.27	0.066

**Dataset 3**				**Dataset 4**			

time [h]	Liver	Kidney	Brain	time [h]	Liver	Kidney	Brain
0	0	0	0	0	0	0	0
0.5	5.75	2.12	0.15	0.5	7.5	2.28	0
2.5	6.05	1.31	0.20	4.5	7.79	1.92	0
20.5	5.50	1.36	0.02	26.5	7.2	1.81	0
71.5	3.57	0.74	0.0	69.5	5.2	1.22	0

**Table 3:** Mass of organs and fraction of blood flow in humans used in the PBPK model

Organ/tissue	Weight [g]	Fraction of blood flow [%]
Lungs	1000	100
Kidney	299	22
Liver	1910	17.5
Brain	1420^a^	11.4
Venous blood	2350 (2.35 L)	100
Rest of body	64371	49.1
Arterial blood	2350 (2.35 L)	100

a - Intravascular compartment (vascular space) or cerebral blood volume (CBV) =3.8%, Extracellular space (ECS) = interstitial space + CBV = 15 - 20%, cellular space = 80 - 95%

### Estimation of the model parameters

The clinical data obtained by WBS was used to obtain the unknown model parameters. This procedure represents the calibration of the model and is equivalent to an optimization problem of finding the model parameters for which the difference between results obtained by model and clinical data are minimal. The difference between model results and clinical data is defined by the objective function. In this work the trust region method has been utilized to minimize the objective function and to obtain the optimal parameters in the PBPK model (35, 34).

In the k-th iteration the objective function is approximated by the first two terms in the Taylor-series expansion inside the trust interval around the point *P*_k_ according to the following equations (37, 36):


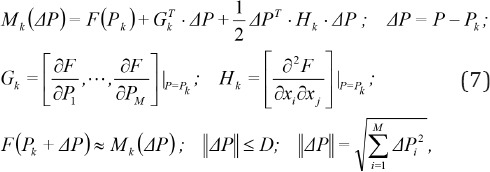


where *F* is the objective function, *M*_k_ is quadratic model function for *F* in the k-th iteration, *G*_k_ and *H*_k_ are gradient and Hessian matrix of the objective function at point *P*_k_, respectively, *D* is radius of the trust region, ||Δ*P*|| is the Euclidian norm of Δ*P*, *G*_k_ is 1×N vector and *H*_k_ is N×N symmetric matrix.

The model function is minimized inside the trust region (sub problem) under constraints that all model parameters are positive and the k+1 point *P*_k+1_ is obtained according to the following relations:


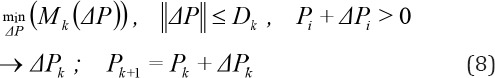


To speed up the numerical procedure, the trust region method has been modified and the Hessisan matrix *H*_k_ is approximated using Broyden-Fletcher-Goldfarb-Shanno (BFGS) Method (20-23).

In each iteration the Hessian matrix is updated using the data from the previous step according to the following relation:


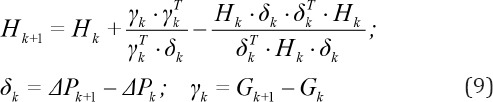


## Results

**Figure 3:**
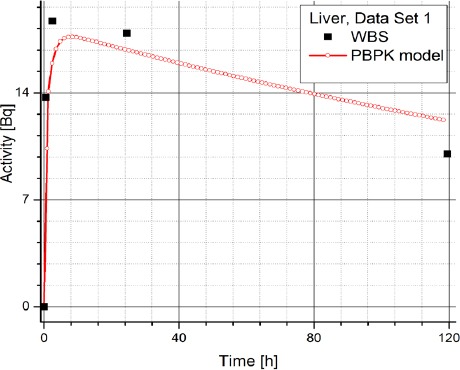
Comparison of time-activity curves in liver for patient 1, obtained by WBS and PBPK model

**Figure 4:**
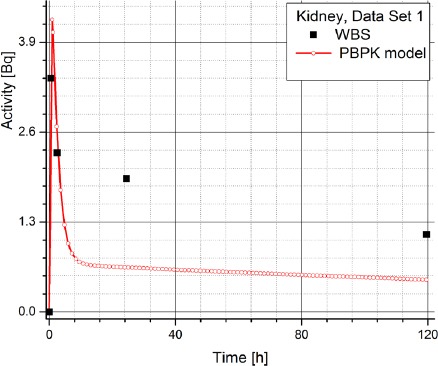
Comparison of time-activity curve in kidney for patient 1, obtained by WBS and PBPK model

**Figure 5:**
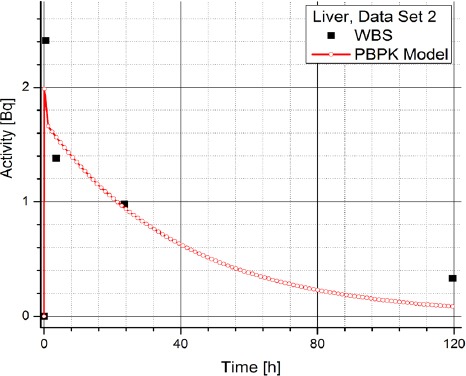
Comparison of time-activity curve in liver for patient 2, obtained by WBS and PBPK model

Table 2 contains the clinical data showing radiopharmaceuticals’ activities in organs (dataset 4-1) which are expressed in counts per second (Bq). The data has been obtained using WBS of patients with NET treated with Lu-177 DOTATATE and it suggests a large inter-individual variability in the patients, which prevents using same model parameters for all the patients. Figures 3 and 4 show the change in time of activity in liver and kidney, respectively, obtained for the dataset 1 by WBS and by the PBPK model. The activity of RLP is expressed in Becquerel [Bq]. Figures 8 - 5, show the similar data to Figures 3 and 4. It is evident that in each case a good agreement is achieved between the model and the WBS data, except for the case of activity in kidneys for dataset 1 where delayed kidney clearance rate is observed for the model results compared to the WBS recordings. While this discrepancy seems prominent, a comparative magnitude of discrepancy is observed in Figure 3 for the activity in liver for dataset 1, except that the activity in liver is by one order of magnitude higher compared to kidneys, so the discrepancy seems smaller.

**Figure 6:**
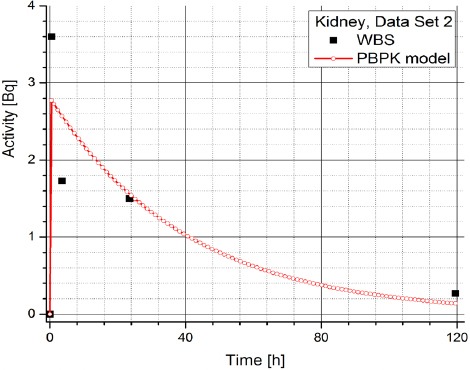
Comparison of time-activity curve in kidney for patient 2, obtained by WBS and PBPK model

**Figure 7:**
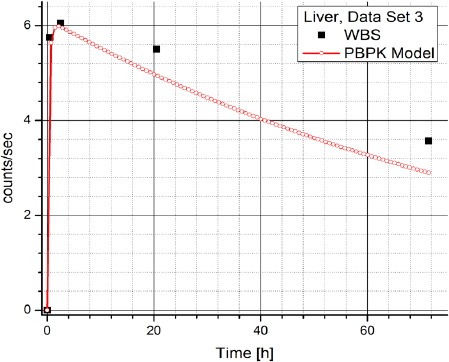
Comparison of time-activity curve in liver for patient 3, obtained by WBS and PBPK model

**Figure 8:**
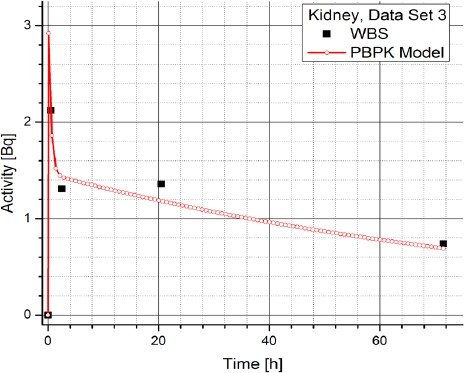
Comparison of time-activity curve in kidney for patient 3, obtained by WBS and PBPK model

## Discussion

The blood flow restricted (perfusion rate limited) type of PBPK model for biodistribution and accumulation in individual organs has been developed for Lu-177 DOTATATE using clinical data obtained by whole body scintigraphy (WBS) in four different patients. This is the first attempt of utilizing scintigraphic data from therapy of patients with NET with Lu-177 peptides in PBPK models. However, the limitation of the study is the low number of the patients available and not applying SPECT results for increased precision in measurements of the activity distributions in the organs.

According to the available data, a PBPK model with seven compartments has been adopted. The model takes into account the four main physiological processes in the organism: Absorption, Distribution, Metabolism and Extraction (ADME). The clearing tissue has been modelled using intrinsic clearance parameter.

The clinical results obtained by WBS for four different patients, see Table 2, show that there is significant difference in the RLPs distribution in different organs in time between different patients. The current results suggest that an approach would be feasible where a PBPK model is calibrated for each patient. This would lead to more accurate extrapolation of the time-activity data when the data on uptake and clearance is not collected for long enough period. The approach also allows for straightforward integration of the time-dependent activity providing increased accuracy and convenience when calculating the absorbed dose in certain tissue/organ. This approach eliminates the problems with inter-patient variability of RLPs distribution due to physiological differences or due to influence of disease and/or therapy. The calibration was carried out using clinical data on biodistribution of RLPs obtained by WBS of the patients. The numerical procedure for calibration of the model parameters has employed the trust-region method. To reduce the required CPU time and to speed up the simulation, the Hessian matrix has been approximated using Broyden-Fletcher-Goldfarb-Shanno (BFGS) method.

From the WBS data it could be observed that for some organs (dataset 1: kidneys; dataset 2: kidneys, liver and brain; dataset 3: kidneys) there is a rapid increase in concentrations of RLPs immediately after administration, which the model was able to reproduce.

The agreement of the model predictions with the data obtained from the WBS is satisfactory, indicating that the PBPK model presented in this study can be used for more accurate calculation of biodistribution of RLPs and more accurate estimation of the absorbed dose in specific tissues/organs in the RLP therapies.

## Conclusion

The study indicates that the PBPK model can be used for more accurate calculation of biodistribution and absorbed doses in patients. This approach is the first attempt of utilizing scintigraphic data in PBPK models, which was obtained during Lu-177 peptide therapy of patients with NET.
